# Bone Health in Patients with Breast Cancer: Recommendations from an Evidence-Based Canadian Guideline

**DOI:** 10.3390/jcm2040283

**Published:** 2013-12-17

**Authors:** Alexander H. G. Paterson, Melissa A. Shea-Budgell

**Affiliations:** 1 Tom Baker Cancer Centre, University of Calgary, 1331-29 Street NW, Calgary, AB T2N 4N2, Canada; 2 CancerControl Alberta, Alberta Health Services, 1331-29 Street NW, Calgary, AB T2N 4N2, Canada; E-Mail: melissa.shea-budgell@albertahealthservices.ca

**Keywords:** bone modifying agent, bisphosphonate, bone metastases, bone mineral density, breast cancer, adjuvant therapy, clinical practice guideline, systematic review

## Abstract

Bone loss is common in patients with breast cancer. Bone modifying agents (BMAs), such as bisphosphonates and denosumab, have been shown to reverse or stabilize bone loss and may be useful in the primary and metastatic settings. The purpose of this review is to provide clear evidence-based strategies for the management of bone loss and its symptoms in breast cancer. A systematic review of clinical trials and meta-analyses published between 1996 and 2012 was conducted of MEDLINE and EMBASE. Reference lists were hand-searched for additional publications. Recommendations were developed based on the best available evidence. Zoledronate, pamidronate, clodronate, and denosumab are recommended for metastatic breast cancer patients; however, no one agent can be recommended over another. Zoledronate or any oral bisphosphonate and denosumab should be considered in primary breast cancer patients who are postmenopausal on aromatase inhibitor therapy and have a high risk of fracture and/or a low bone mineral density and in premenopausal primary breast cancer patients who become amenorrheic after therapy. No one agent can be recommended over another. BMAs are not currently recommended as adjuvant therapy in primary breast cancer for the purpose of improving survival, although a major Early Breast Cancer Cooperative Trialists’ Group meta-analysis is underway which may impact future practice. Adverse events can be managed with appropriate supportive care.

## 1. Introduction

Optimization of bone health is an important aspect of care to consider for patients with a breast cancer diagnosis at any stage. In patients with primary breast cancer, treatment associated bone loss can occur either with adjuvant endocrine therapy (e.g., aromatase inhibitors), or in patients with a reduction of ovarian function due to surgery or chemotherapy. Bone loss in this setting is due to enhanced bone turnover secondary to estrogen decline [1]. Other factors, unrelated to breast cancer, that may also contribute to the risk of bone loss, include age (>65 years), race (Caucasian), low body mass index (<20 kg/m^2^), family and personal history of fractures, menopausal status, oral corticosteroid use, history of osteoporosis, and smoking [2]. In the primary breast cancer setting, bone loss can present (as is the case with osteopenia or osteoporosis) as bone fractures, bone pain, and/or impaired mobility. The rate of vertebral fractures is five times higher among women with breast cancer, compared to the general population [3]. As such, there is an impact on overall quality of life for these patients.

Bone health can also be compromised in the metastatic breast cancer setting as a result of malignant cells stimulating bone resorption via osteoclasts; this breakdown of bone can stimulate further tumour growth, leading to an increased risk of skeletal related events such as pain, vertebral and long bone fracture (with associated morbidity), spinal cord compression, and hypercalcemia [4,5]. Among patients with metastatic breast cancer, the incidence of bone metastases at some point during the course of the disease is approximately 73% [6].

To date, efforts to optimize bone health have focused on bisphosphonates and biological agents, such as denosumab. Although the effectiveness of bone modifying agents (BMAs) in improving bone health has been established, uncertainties still remain (e.g., ideal time to initiate therapy, duration of therapy, modification of therapy, *etc*.). The purpose of this paper is to provide clear evidence-based strategies for the management of bone loss and symptoms of bone loss in patients with a breast cancer diagnosis. Specifically, the following questions are addressed: (1) In patients with metastatic breast cancer, when should bone modifying agents (BMAs) be used? Which BMAs should be considered and for how long? Should the BMA be switched after a skeletal-related event (SRE) or documentation of disease progression in bone? (2) In patients with primary breast cancer, how and when should fracture risk be assessed? Is there a role for BMAs in these populations and, if so, which agents should be considered and for how long? How should treatment with BMAs be monitored for effectiveness? (3) Should BMAs be used as adjuvant therapy to improve breast cancer-related outcomes? (4) What adverse events can occur with BMA use and should be disclosed to patients? What is the frequency of these adverse events with the different agents and schedules of administration? How should these adverse events be managed?

## 2. Methodology

### 2.1. Guideline Development

The review process for this guideline was developed, based on: (1) The National Institute for Health and Clinical Excellence (NICE) overview of clinical guideline development for stakeholders, the public, and the National Health Service (NHS, England) [7]; (2) Cummings and Rivara’s methodology on reviewing manuscripts for Archives of Pediatrics & Adolescent Medicine (2002) [8]; and (3) The Appraisal of Guidelines for Research and Evaluation (AGREE) collaboration [9]. With that methodological foundation, the guideline recommendations were drafted by a medical oncologist from the Tom Baker Cancer Centre, Calgary, Alberta, with support from a research methodologist and an expert panel of physicians from the Province of Alberta Breast Tumour Team. Members of the Breast Tumour Team include medical oncologists, radiation oncologists, surgeons, pathologists, psychosocial oncologists, nurses, and pharmacists. The evidence base for the guideline was informed by a systematic review of the literature. The literature will be periodically reviewed and the guideline will be updated as new or compelling evidence is identified.

### 2.2. Literature Search Strategy

A systematic search for relevant literature related to breast cancer and bone health was conducted of MEDLINE (1996 to 2012 July) and EMBASE (1996 to 2012 July). The search included the terms “breast neoplasm” and “zoledronic acid” or “zoledronate” or “clodronate” or “clodronic acid” or “alendronate” or “alendronic acid” or “pamidronate” or “pamidronic acid” or “ibandronate” or “ibandronic acid” or “denosumab”. The search was limited to randomized controlled trials and meta-analyses. Reference lists of key clinical trials were also hand-searched for additional literature. Guidelines on this topic were identified from a search of the National Guidelines Clearinghouse database, as well as individual guideline developers’ websites.

Articles were selected for inclusion in the review of the evidence if they reported outcomes related to survival, bone mineral density or skeletal related events, or adverse events. Indirect evidence with other oncologic-patient populations treated with BMAs was also deemed relevant if insufficient primary evidence was available. Articles were excluded from the review of the evidence if they were qualitative or descriptive studies, opinion papers, letters, or editorials. Due to a lack of translation services, non-English language articles were excluded from the review of the evidence.

## 3. Results and Discussion

### 3.1. Results of Literature Review

A total of 57 published articles were deemed relevant to inform the role of BMAs in breast cancer. Twenty-nine publications focused on the use of BMAs in the prevention of treatment-related bone loss in patients with primary breast cancer (Table 1); thirteen publications examined the efficacy of BMAs in preventing skeletal related events (SREs) in patients with metastatic breast cancer (Table 2); and fifteen publications looked at the use of BMAs in the adjuvant setting in patients with primary breast cancer (Table 3).

The literature search identified seven clinical practice guidelines that provided recommendations on the use of bisphosphonates in the setting of breast cancer; these guidelines were developed by: American Society of Clinical Oncology (ASCO) [10,11], the National Comprehensive Cancer Network (NCCN) [12], Cancer Care Ontario (CCO) [13], the British Columbia Cancer Agency (BCCA) [14], the International Society of Geriatric Oncology (ISGO) [15], the European Expert Panel (EEP) [16], and Cancer Care Australia [17]. Following the initial search, an additional Canadian guideline, BONUS 6, was published by a panel of experts in the field [18] and ASCO released an updated version of their guideline [11]. The World Health Organization’s Fracture Risk Assessment Tool (FRAX) [19] was also identified by the literature search and was included in the review in order to establish patients at risk of fracture.

**Table 1:** Evidence on the use of bone modifying agents (BMAs) in patients with treatment-related bone loss from breast cancer.

Author, year	Intervention	Administration	Patients (*N*)	Outcome(s)
BMAs in Patients Undergoing Endocrine Therapy				
Safra T. 2011 [20]	ZOL Control	4 mg IV q6 m	47 43	BMD
van Poznak C. 2010 [21]	RIS Placebo	35 mg/w oral	77 77	BMD, bone turnover
Hines S. 2010 [22]	ZOL	4 mg IV q6 m	60	BMD
Gnant M. 2009 [22,23,24,25]	ZOL No treatment	4 mg IV q6 m	205 199	BMD
Ellis G. K. 2009 [23,24]	DEN Placebo	60 mg SC q6 m	127 125	BMD
Hines S. L. 2009 [25]	RIS Placebo	35 mg/w oral	106 106	BMD
Cohen A. 2008 [26]	ALE Placebo	70 mg/w oral	6 5	BMD
Lester J. E. 2008 [27]	IBA Placebo	150 mg oral q4 w	25 25	BMD, bone turnover, fractures
**BMAs in Patients Undergoing Chemotherapy**				
Kim J. 2011 [28]	ZOL No treatment	4 mg IV q6 m	55 55	BMD, bone turnover
McCloskey E. 2010 [29]	CLO Placebo	1600 mg/d oral	419 432	BMD, bone turnover
Hershman D. 2010 [30,31]	ZOL Placebo	4 mg IV q6 m	50 53	BMD, bone turnover
Greenspan S. 2008 [32,33]	RIS Placebo	35 mg/w oral	43 44	BMD, bone turnover
Fuleihan G. H. 2005 [34]	PAM Placebo	60 mg IV q3 m	21 19	BMD, bone turnover
Delmas P. D. 1997 [35]	RIS Placebo	30 mg/d oral × 14 then q12 w	27 26	BMD, bone turnover
Saarto T. 1997 [36,37]	CLO Placebo	1600 mg/d oral	44 49	BMD
**BMAs (Upfront *vs*. Delayed) in Patients Undergoing Endocrine Therapy**				
Brufsky A. M. 2009 [38,39,40,41]	ZOL upfront ZOL delayed	4 mg IV q6 m	300 300	BMD, bone turnover
Eidtmann H. 2010 [42,43,44]	ZOL upfront ZOL delayed	4 mg IV q6 m	532 533	BMD, bone turnover, fractures
Brufsky A. M. 2008 [45]	ZOL upfront ZOL delayed	4 mg IV q6 m	832 833	BMD, bone turnover
Llombart A. 2012 [46,47]	ZOL upfront ZOL delayed	4 mg IV q6 m	252 270	BMD
Hines S. L. 2009 [48]	ZOL upfront ZOL delayed	4 mg IV q6 m	197 198	BMD

Note: ZOL—zoledronate; RIS—risedronate; PAM—pamidronate; CLO—clodronate; IBA—ibandronate; DEN—denosumab; ALE—alendronate; q—every; m—months; w—weeks; d—day; BMD—bone mineral density; SC—subcutaneous; IV—intravenous.

**Table 2:** Evidence on the use of BMAs in patients with metastasis-related bone loss from breast cancer.

Author, year	Intervention	Administration	Patients (*N*)	Outcome(s)
Body J. J. 2010 [49]	DEN ZOL/PAM/IBA	30/120/180 mg SC q4 w as per label	127 85	SRE, bone turnover
Stopeck A. 2010 [50]	ZOL DEN	4 mg IV q4 w 120 mg SC	2046 (total)	SRE, OS, progression in bone
Body J. J. 2007 [51]	IBA ZOL	50 mg/d 4 mg IV q4 w	137 138	bone turnover, bone pain
Kohno N. 2005 [52]	ZOL Placebo	4 mg IV (15-min) q4 w	114 114	SRE
Tripathy D. 2004 [53]	IBA Placebo	20 or 50 mg/d oral	148/144 143	SRE, bone turnover
Rosen L. S. 2004 [54]	ZOL PAM	4 mg IV q3–4 w 90 mg IV q3–4 w	372 140	SRE
Body J. J. 2003 [55]	IBA Placebo	2 or 6 mg q3–4 w 2 mg q3–4 w	154/154 158	SRE
Lipton A. 2000 [56]	PAM Placebo	90 mg IV q3–4 w × 24 cycles	367 384	SRE, bone turnover, bone pain
Kristensen B. 1999 [57]	CLO No treatment	800 mg/d oral	49 51	SRE, bone pain
Theriault R. 1999 [58]	PAM Placebo	90 mg IV q4 w × 24 cycles	182 189	SRE, bone pain
Hortobagyi G. 1998 [59]	PAM Placebo	90 mg IV q3–4 w	185 197	SRE, bone turnover, bone pain
Conte P. F. 1996 [60]	PAM No treatment	45 mg IV q3 w	143 152	progression in bone, bone pain
Paterson A. 1993 [61]	CLO Placebo	1600 mg/d oral	85 88	SRE, bone pain, OS

Note: ZOL—zoledronate; PAM—pamidronate; CLO—clodronate; IBA—ibandronate; DEN—denosumab; w—weeks; d—day; SC—subcutaneous; IV—intravenous; SRE—skeletal related events/complications; OS—overall survival.

**Table 3:** Evidence on the use of BMAs as adjuvant therapy in patients with primary breast cancer.

Author, year	Intervention	Administration	Patients (*N*)	Outcome(s)
Paterson A. 2012 [62]	CLO Placebo	1600 mg/d oral	1662 1661	DFS
Gnant M. 2011 [63,64,65,66]	ZOL No treatment	4 mg IV q6 m	899 904	DFS, OS
Coleman R. E. 2011 [67,68]	ZOL No treatment	4 mg IV × 6 q3–4 w then q3–6 m	3360 (total)	DFS, OS
Aft R. 2010 [69]	ZOL No treatment	4 mg IV q3 w	60 59	DFS, RFS, OS
Kristensen B. 2008 [70]	PAM No treatment	150 mg/d × 2 oral	460 493	SRE, OS
Diel I. J. 2008 [71,72]	CLO No treatment	1600 mg/d oral	157 145	DFS, OS
Powles T. 2002 [73,74]	CLO Placebo	1600 mg/d oral	530 539	MFS, OS
Saarto T. 2001 [75,76]	CLO No treatment	1600 mg/d oral	139 143	DFS, OS

Note: ZOL—zoledronate; PAM—pamidronate; CLO—clodronate; IV—intravenous; w—weeks; d—day; m—months; DFS—disease-free survival; OS—overall survival; RFS—recurrence-free survival; MFS—metastasis-free survival; SRE—skeletal related events.

### 3.2. Discussion

Bone loss can occur in breast cancer due to treatment, especially estrogen suppressing therapy and certain chemotherapies, or due to the disease itself [1,4]; however, bone modifying agents such as bisphosphonates and denosumab offer patients with breast cancer a way to treat and potentially prevent bone loss. The following discussion of the literature will highlight trials demonstrating the effectiveness and safety of these bone modifying agents and describe data on less established areas, such as the timing of therapy, the duration of therapy, and the use of therapy as an adjuvant to prolong survival.

#### 3.2.1. Treatment-Related Bone Loss

The effectiveness of bisphosphonates as bone modifying agents has been well established. Trials employing the use of zoledronate, risedronate, alendronate, ibandronate, clodronate, and pamidronate in patients receiving aromatase inhibitors or chemotherapy have demonstrated that these agents increase bone mineral density and decrease bone turnover and the risk of fractures. Zoledronate (4 mg IV, every six months) significantly increased bone mineral density at the hip and spine (+3.9% and +4.0%, respectively, *vs.* baseline) in early stage premenopausal patients with ovarian suppression (*n* = 404) at five years follow-up, as compared to placebo which incurred significant losses at these sites (−4.1% and −6.3%, respectively, *vs.* baseline) [63,64,65,66]. Others have observed similar changes in bone mineral density with the use of zoledronate among premenopausal patients [30,31]. Pamidronate (60 mg IV, every three months), but not risedronate (35 mg per week, orally), was able to achieve a significant 1.9% increase in bone mineral density at the spine, but not at hip, among premenopausal patients undergoing chemotherapy [25,34]. No significant difference in the rate of fractures has been observed among the premenopausal group in any of these studies. There have been mixed results regarding the markers of bone turnover (*i.e.*, N-terminal telopeptide (NTX), C-terminal cross-linked telopeptide of type I collagen (CTX-I), N-terminal propeptide of type I collagen (P1NP), and bone alkaline phosphatase (AP)) [63,64,65,66,77].

Among post-menopausal women receiving aromatase inhibitors, a variety of bisphosphonates have been tested and show similar improvements in bone loss. Zoledronate (4 mg IV, every six months) was shown to maintain or significantly increase (+2.66% *vs.* baseline) bone mineral density at the spine [20,22]. Similarly, in the Study of Anastrozole with the Bisphosphonate Risedronate (SABRE) and Risedronate’s Effect on Bone in Women with Breast Cancer (ReBBeCa) trials, risedronate demonstrated significantly better maintenance or increases in bone mineral density, *vs.* placebo (2.5%–2.9% higher than placebo for hip and 1.6%–4.0% higher than placebo for spine); significant improvements in NTX, CTX-I, PINP, and bone AP, indicating a reduction in bone turnover, were also observed [21,32,33]. The Anastrozole-Induced Bone Loss (ARIBON) trial likewise demonstrated improvements in bone mineral density at the hip and spine (4.5% higher than placebo for hip and 6.2% higher than placebo for spine) with ibandronate (150 mg orally, every 28 days); in addition, ibandronate improved serum levels of NTX, CTX-I, bone AP, as well as T-score [27]. Oral clodronate (1600 mg per day) also led to a significant improvement in bone mineral density at the hip, spine, and femoral neck among postmenopausal patients [29,55,73]. Finally, alendronate has also demonstrated efficacy among postmenopausal patients in maintaining or increasing bone mineral density at the spine and femoral neck [26]. To date, no trials comparing one bisphosphonate with another have been conducted in the setting of treatment-related bone loss; bisphosphonates are generally thought to be comparable in terms of efficacy and the decision to use one agent over another is often related to route of administration or other factors that could affect compliance.

The ratio of receptor osteoprotegerin (OPG) to receptor activator of nuclear factor-kappa B ligand (RANKL) plays a role in osteoclastogenesis: When RANKL levels are high, bone loss will occur. However, by altering the ratio in favor of OPG by inhibiting RANKL, bone loss can be prevented [78]. Therefore, biological agents that inhibit RANKL may prevent bone loss due to treatment or metastases. Denosumab, a RANKL inhibitor, has demonstrated efficacy among postmenopausal patients receiving aromatase inhibitors. As compared to placebo, denosumab (60 mg SC, every six months) significantly increased bone mineral density of the hip (+4.7% *vs.* placebo), spine (+7.6% *vs.* placebo), wrist (+6.1% *vs.* placebo), and femoral neck (+3.6% *vs.* placebo) [23,24]. In this trial, denosumab also significantly improved serum levels of CTX (−91% *vs.* +9% for placebo) and PINP (−29% *vs.* −2% for placebo), but did not significantly improve the rate of fractures.

Recommended agents for the prevention and management of bone loss include zoledronate (IV 4 mg over no less than 15 min every 6–12 months) and denosumab (60 mg SC every 6 months). Any oral bisphosphonate is acceptable as well, including clodronate (1600 mg per day), risedronate (35 mg per day), or alendronate (70 mg per day). The route of administration should be left to the discretion of the treating physician, taking into account compliance with treatment, cost of treatment, and patient preference.

The timing of therapy (upfront *vs.* delayed) has been evaluated in several trials, among postmenopausal patients receiving aromatase inhibitors. The Zometa-Femara Adjuvant Synergy Trials (Z-FAST, ZO-FAST, and EZO-FAST) [38,39,40,41,42,43,44,46,47] and a National Cancer Institute (NCI) trial [48] compared zoledronate upfront (*i.e.*, immediately) with delayed *(i.e.*, following a fracture or a decrease in bone mineral density). Immediate zoledronate (4 mg IV, every six months) significantly increased bone mineral density at both the hip (5.4%–6.7% higher than delayed) and the spine (8.6%–9.3% higher than delayed) in the ZO-FAST and Z-FAST trials, as well as in meta-analysis of the combined data (hip: 3.4% higher than delayed; spine: 5.1% higher than delayed) [45]. The EZO-FAST and NCI trials also reported significant improvements in bone mineral density at the hip and spine. Where reported, there were no significant differences in T-scores, fracture rates, or overall survival (OS) for any of these studies.

#### 3.2.2. Metastasis-Related Bone Loss

Bone loss can occur in metastatic breast cancer as a result of malignant cells stimulating bone resorption, which in turn can lead to further tumour growth [4]. Bone modifying agents may, therefore, be able to reverse or stabilize bone loss in patients with bone metastases. In a trial comparing clodronate (1600 mg per day, orally) with placebo, clodronate was shown to reduce the incidence of skeletal-related events (SREs) among patients with bone metastases from breast cancer [61]. Zoledronate (4 mg IV, every four weeks) was shown to significantly decrease the fracture rate (25.4% *vs.* 38.9% for placebo) and lower the incidence of one or more SREs (29.8% *vs.* 49.6% for placebo) among breast cancer patients with bone metastases [52]. Likewise, pamidronate (90 mg IV every three to four weeks for 24 cycles) significantly decreased the fracture rate (40% *vs.* 52% for placebo), the level of bone AP (−33% *vs.* +5% for placebo), and the incidence of one or more SREs (51% *vs.* 64% for placebo) [56]. Similar significant positive changes in the fracture rate and incidence of SREs were observed for ibandronate (2 or 6 mg every three to four weeks) [55]. Zoledronate, pamidronate, and ibandronate were then compared with denosumab and demonstrated similar efficacy, in terms of OS, at six months, among breast cancer patients with bone metastases (85% for denosumab *vs.* 81% for bisphosphonates) [49]. Monthly SC denosumab (120 mg) has been shown to be superior to monthly IV zoledronate in delaying time to first SRE and cumulative SREs [50].

Bone modifying agents are recommended for patients with breast cancer with evidence of bone metastases. The presence of non-bone metastases is not a clear indication for the use of bone modifying agents; however bone modifying agents can be used when osteopenia (*i.e.*, T-score between −1.0 and −2.5) or osteoporosis (*i.e.*, T-score less than −2.5) is present. The FDA has approved zoledronic acid (5 mg once every 2 years) for the prevention of postmenopausal osteoporosis [79]; however, recommendations cannot be made favoring one agent over another. Acceptable agents and dosing regimens for bone metastases include zoledronate (4 mg IV over no less than 15 min every 4 weeks), pamidronate (90 mg IV over no less than 2 h every 3 to 4 weeks), clodronate (1600 mg per day orally), and denosumab (120 mg SC every four weeks). There are several advantages and limitations to the different agents and routes of administration. The route of administration should be left to the discretion of the treating physician, taking into account compliance with treatment, cost of treatment, and patient preference.

#### 3.2.3. Bone Modifying Agents as Adjuvant Therapy

The use of bone modifying agents as an adjuvant to standard therapy has also been investigated. The Austrian Breast and Colorectal Cancer Study Group Trial 12 (ABCSG-12) looked at the effect of adding zoledronate (4 mg IV, every six months) to endocrine therapy in early stage premenopausal patients with ovarian suppression (*n* = 404); after a median follow-up of 47.8 months, the trial demonstrated that zoledronate provided a gain in DFS (90.8% for endocrine therapy alone *vs*. 94.0% for endocrine therapy with zoledronate), but not a reduction in the risk of death (hazard ratio, 0.60; 95% CI, 0.32 to 1.11; *p* = 0.11). The addition of zoledronate resulted in a relative reduction of 35% in the risk of recurrence (hazard ratio, 0.65; 95% CI, 0.46–0.92; *p* = 0.01) [63,64,65,66]. Clodronate (1600 mg per day, orally) taken over a duration of two years significantly improved OS (81.5% *vs.* 76.1% for placebo) and reduced the incidence of bone metastases (9.6% *vs.* 13.5% for placebo) among patients with primary operable breast cancer [73,74]. However, pamidronate (150 mg per day, orally) did not improve OS or the rate of bone metastases, *vs.* placebo [70]. Similarly, among patients with breast cancer (stages II–III) in the Adjuvant Zoledronic Acid to Reduce Recurrence (AZURE) trial, zoledronate (4 mg every 3–4 weeks for six doses, before or after surgery, followed by 4 mg every 3 months for eight doses and then every 6 months for five doses) did not improve DFS (77% *vs.* 77%) or OS (85.4% *vs.* 83.1%) as compared to standard therapy; however, a sub-group analysis of postmenopausal patients revealed a significant improvement with treatment [67,68]. The National Surgical Adjuvant Breast and Bowel Project (NSABP) B-34 trial recently published the 7.5 year (median 90.7 months follow up) analysis of survival data among early breast cancer patients treated with oral clodronate (1600 mg per day) for three years or placebo, with similar findings of a sub-group advantage in postmenopausal patients for distant metastases-free interval in the treated patients [62]. Overall, evidence for the use of bisphosphonates as adjuvant therapy for prevention of bone metastases in women with breast cancer is undergoing review and meta-analysis to determine if benefit exists in subgroups such as postmenopausal patients. Ongoing research includes the Denosumab as Adjuvant Treatment for Women with High Risk Early Breast Cancer Receiving Neoadjuvant or Adjuvant Therapy (D-CARE) trial, which randomizes patients with bone metastasis to denosumab or placebo and follows patients for the main outcomes of DFS and bone metastasis-free survival [80]. Until such data are available, the use of bone modifying agents is not recommended outside of a clinical trial for patients with breast cancer as a standard adjuvant therapy to improve recurrence or survival rates.

#### 3.2.4. Adverse Events

Adverse events associated with bisphosphonate use (oral or intravenous) and denosumab are typically mild and manageable, but include arthralgia, fever, thrombosis, bone pain, fatigue/tiredness, nausea, and gastrointestinal symptoms [20,21,24,25,26,27,29,36,81]. Patients undergoing therapy with bone modifying agents should be monitored throughout therapy for changes in renal function (*i.e*., creatinine clearance, serum calcitriol, serum calcium) [9,82,83,84,85]. In addition, patients with poor dental hygiene or poor dental health may be at increased risk of osteonecrosis of the jaw, especially those patients receiving potent IV BMAs [86,87]; therefore, patients should undergo preventive dentistry before starting treatment with a bone modifying agent and dentition should be monitored during therapy [9]. Furthermore, dental extractions should be avoided if possible and carried out by an experienced dentist. The Zoledronic Acid for Prolonged Treatment of Patients with Bone Metastases from Breast Cancer (ZOOM) trial recently compared 4-weekly and 12-weekly zoledronic acid (4 mg) in patients with breast cancer with one or more bone metastases (*n* = 425) and showed that 12-weekly treatment was not inferior to 4-weekly treatment. However, the trial was underpowered to identify clinically significant differences in grade 3–4 toxicity (*i.e.*, bone pain, nausea, and asthenia), renal adverse events, or osteonecrosis of the jaw. N-Terminal telopeptide concentration did change significantly from baseline to 12 months in the 12-weekly group *vs.* the 4-weekly group (median 12.2% *vs.* 0.0%, respectively; *p* = 0.011) [88].

Recent observations have raised the possibility of atypical fractures occurring in patients on long-term BMAs [89]; among patients with atypical fracture, the prevalence of bisphosphonate use was 90% [90]. Ongoing surveillance for atypical and minimal trauma fractures is warranted in patients undergoing therapy with BMAs.

## 4. Conclusions

### 4.1. Summary of Recommendations on Treatment-Related Bone Loss

Baseline bone mineral density (BMD) testing and fracture risk assessment is recommended for patients with primary breast cancer for whom therapy with agents that suppress ovarian function is given, including premenopausal women with premature ovarian failure or ovarian suppression with luteinizing hormone releasing hormone analogue (LHRHA) and postmenopausal women on aromatase inhibitors (AIs). BMD testing in other postmenopausal women with primary breast cancer is recommended according to the Canadian Osteoporosis screening guidelines using a BMD cut off of T-score <−2.5 [91]. BMD is calculated using a dual-energy X-ray absorptiometry (DEXA) scan. Fracture risk should be assessed using the World Health Organization Fracture Risk Assessment Tool [19]. Repeat BMD testing should be performed as follows, in patients for whom pharmacotherapy with bone modifying agents is deemed to be not beneficial: Every five years in low risk patients (10-year risk <10% based on FRAX score) and every one to three years in moderate risk patients (10-year risk 10%–20% based on FRAX score).

BMAs should be considered for the following patients with primary breast cancer: Premenopausal OR postmenopausal at high risk (*i.e.*, 10-year fracture risk >20% OR prior fragility fracture of hip or spine OR more than one fragility fracture) and postmenopausal at moderate risk (*i.e.*, 10-year fracture risk 10%–20%) OR a T-score lower than −2.0, AND undergoing aromatase inhibitor therapy for breast cancer. As per the Canadian Osteoporosis guidelines [91], exercise, adequate calcium (1200 mg per day total, diet plus supplements) intake, and vitamin D (1000 IU per day) supplementation are also recommended. For patients with primary breast cancer, no recommendations can be made favoring one bone modifying agent over another. Acceptable agents and dosing regimens for bone loss include: zoledronate (intravenous 4 mg over no less than 15 min, every 6–12 months), any oral bisphosphonate, denosumab (subcutaneous 60 mg, every 6 months). The route of administration should be left to the discretion of the treating physician, taking into account compliance with treatment, cost of treatment, and patient preference. There is no data on the optimal duration of therapy with bone-modifying agents for patients with primary breast cancer with treatment-related bone loss. Most randomized controlled trials have used durations of two to three years and none have compared one time period with another.

In patients with primary breast cancer undergoing therapy with a bone modifying agent, BMD can be checked every two years. However, in patients with osteopenia, BMD should be checked annually.

### 4.2. Summary of Recommendations on Metastasis-Related Bone Loss

In patients with metastatic breast cancer, bone modifying agents (BMAs) are recommended upon confirmation of bone metastases; the presence of non-bone metastases is not an indication for the use of bone modifying agents.

For patients with breast cancer with bone metastases, no recommendations can be made favoring one agent over another. Acceptable agents and dosing regimens for bone metastases include: zoledronate (intravenous 4 mg over no less than 15 min, monthly), pamidronate (intravenous 90 mg over no less than 2 h, monthly), clodronate (oral 1600 mg, daily) and denosumab (subcutaneous SC 120 mg, monthly). There are advantages and limitations to the different agents and routes of administration. The agent and route of administration should be left to the discretion of the treating physician, taking into account compliance with treatment, cost of treatment, and patient preference. BMAs should be continued in patients with breast cancer with bone metastases until there is evidence of a substantial decline in performance status. In the case of progressive disease, a change in systemic anti-cancer treatment is warranted with continuation of BMAs.

In patients with breast cancer with bone metastases who have experienced a skeletal-related event (SRE) or progression in bone metastases, switching from one bisphosphonate to another is currently not recommended, since no double-blind data are available to support this strategy. However, some oncologists are switching from a bisphosphonate to denosumab at progression, based on proven better efficacy [53].

### 4.3. Summary of Recommendations on Adjuvant Therapy

Outside of a clinical trial, bone modifying agents are not recommended at this time for patients with primary breast cancer, as a standard adjuvant therapy to improve recurrence or survival rates. The Early Breast Cancer Cooperative Trialists’ Group meta-analysis of adjuvant bisphosphonate trials is ongoing and may impact future practice.

### 4.4. Summary of Recommendations on Adverse Events

Patients undergoing therapy with BMAs should be aware that the most common adverse events include nausea, fatigue, arthralgia, back pain, pyrexia, bone pain, vomiting, anemia, diarrhea, dyspnea, extremity pain, and constipation. Patients should also be monitored for changes in renal function (*i.e.*, creatinine clearance). In addition, patients with poor dental hygiene or poor dental health may be at increased risk of osteonecrosis of the jaw and should undertake preventive dentistry before starting treatment with a bone modifying agent and avoid dental extraction. Adverse events should be managed with appropriate supportive care. Denosumab does not require specific monitoring of renal function but as with bisphosphonates, patients should be monitored for rare instances of symptomatic hypocalcemia.
